# Fetal Adrenal Gland Biometry and Middle Adrenal Artery Doppler in Pregnancies Presenting with Preterm Labor: A Prospective Case–Control Study

**DOI:** 10.3390/jcm15031192

**Published:** 2026-02-03

**Authors:** Belgin Savran Üçok, Özgür Volkan Akbulut, Sadun Sucu, Mustafa Bağcı, İbrahim Buğra Bahadır, Kadriye Yakut Yücel

**Affiliations:** 1 Department of Obstetrics and Gynecology, Ankara Etlik City Hospital, 06170 Ankara, Türkiye; 2 Perinatology Clinic, Ankara Etlik City Hospital, 06170 Ankara, Turkey; akbulutvolkan@yahoo.com (Ö.V.A.); ibbahadir@hotmail.com (İ.B.B.); yakutkadriye@hotmail.com (K.Y.Y.); 3 Perinatology Clinic, Eskişehir City Hospital, 26080 Eskişehir, Turkey; drssucu@gmail.com; 4 Van Regional Training and Research Hospital, 65300 Van, Turkey; mustafabagci@outlook.com.tr

**Keywords:** preterm labor, preterm birth, fetal adrenal gland, fetal zone, Doppler ultrasound, middle adrenal artery, cervical length

## Abstract

**Objective**: This study aimed to compare fetal adrenal gland volume (AGV), fetal zone (FZ) depth, and middle adrenal artery pulsatility index (MAA-PI) between pregnancies presenting with preterm labor and gestational age-matched asymptomatic controls, and to evaluate size-adjusted adrenal metrics (corrected AGV [cAGV] and fetal zone–total gland depth ratio) in relation to gestational age at delivery and neonatal outcomes. **Methods**: This prospective analytical cross-sectional (case–control) study included 60 singleton pregnancies (30 with preterm labor and 30 asymptomatic controls) evaluated at a tertiary perinatology unit between 24 + 0 and 36 + 6 weeks’ gestation. Transvaginal cervical length and transabdominal fetal adrenal measurements (AGV, FZ depth, and MAA-PI) were obtained at enrollment. Estimated fetal weight (EFW) at the index scan was retrieved, and corrected AGV (cAGV = AGV/EFW) and fetal zone–total gland depth ratio were calculated. Outcomes were gestational age at delivery, birthweight, Apgar scores, and neonatal intensive care unit (NICU) admission. Nonparametric group comparisons and Spearman correlations were used. **Results**: Gestational age at ultrasound was identical between groups (median 31 + 6 weeks). Compared with controls, the preterm labor group had shorter cervical length (12.5 vs. 33.5 mm, *p* < 0.001), higher AGV (1.53 vs. 1.08 cm^3^, *p* < 0.001) and FZ depth (7.45 vs. 5.30 mm, *p* < 0.001), and lower MAA-PI (1.11 vs. 1.46, *p* < 0.001). EFW at the index scan did not differ between groups (*p* = 0.900). Corrected AGV (cAGV) was higher in the preterm labor group (0.87 (0.76–1.06) vs. 0.59 (0.51–0.70), *p* < 0.001), and the fetal zone–total gland depth ratio was higher (0.328 (0.312–0.346) vs. 0.263 (0.241–0.278), *p* < 0.001). The preterm labor group delivered earlier (33 + 0 vs. 36 + 2 weeks, *p* < 0.001), had lower birthweight (1875 vs. 3188 g, *p* < 0.001), and more frequent NICU admission (50.0% vs. 6.7%; odds ratio 14.0, 95% CI 2.82–69.56; *p* < 0.001). Within the preterm labor group, gestational age at delivery correlated positively with cervical length (ρ = 0.900) and MAA-PI (ρ = 0.770) and negatively with AGV (ρ = −0.770) and FZ depth (ρ = −0.733), all *p* < 0.001; correlations were stronger for cAGV (ρ = −0.953, *p* < 0.001). **Conclusions**: Enlarged fetal adrenal gland volume and fetal zone depth together with reduced middle adrenal artery pulsatility index are associated with preterm labor and earlier delivery. Size-adjusted adrenal metrics (cAGV and fetal zone–total gland depth ratio) remained significantly different between groups, supporting these measures as potential adjuncts for risk stratification at presentation.

## 1. Introduction

Preterm birth is a major driver of neonatal morbidity and mortality and remains a global public health priority [1,2]. In clinical practice, many women present with symptoms suggestive of preterm labor, yet only a subset will deliver soon after presentation. This uncertainty can lead to unnecessary admissions and treatments, while delayed recognition in truly high-risk pregnancies may limit opportunities to optimize perinatal care. Current recommendations emphasize transvaginal cervical length measurement and selective biochemical testing, but overall prediction of spontaneous preterm birth in symptomatic women is still suboptimal [3].

The fetal hypothalamic–pituitary–adrenal axis is a key component of the physiological pathway to parturition. The fetal adrenal gland, and especially its large inner fetal zone, responds to fetal stress and participates in steroidogenesis relevant to the initiation of labor. Sonographic adrenal enlargement and fetal zone expansion have therefore been proposed as markers of an activated fetal stress response preceding spontaneous preterm birth.

In symptomatic cohorts, Turan and colleagues demonstrated that fetal adrenal gland volume and fetal zone enlargement could identify women at risk of delivery within days and, in some reports, performed favorably compared with cervical length [4,5]. Their subsequent methodological work supported the feasibility of both 2D and 3D adrenal volumetry and suggested that corrected adrenal volume and fetal zone metrics may improve prediction [6]. Other studies have also reported useful performance of fetal adrenal biometry and adrenal artery Doppler indices for short-term prediction of delivery, comparable to cervical length in some settings [7,8]. Multimodal strategies combining adrenal measures with elastography, biochemical tests, or both have further supported the clinical plausibility of adrenal-based risk stratification [9,10,11].

Compared with adrenal biometry, data on fetal adrenal artery Doppler remain more limited, and standardization has been challenging. A longitudinal study established zone-specific reference ranges for inferior and middle adrenal artery Doppler indices and highlighted zonal differences in adrenal perfusion [12]. Feasibility studies have also demonstrated that fetal adrenal arteries can be visualized and interrogated with modern ultrasound technology [13]. Against this background, the present study aimed (1) to compare AGV, fetal zone depth, and MAA-PI between pregnancies presenting with preterm labor and gestational age-matched asymptomatic controls, and (2) to evaluate associations between these markers and delivery and neonatal outcomes [14].

## 2. Materials and Methods

### 2.1. Study Design and Setting

This prospective analytical cross-sectional (case–control) study was conducted at a tertiary perinatology unit between 1 September 2023 and 1 September 2024. The manuscript follows the Strengthening the Reporting of Observational Studies in Epidemiology (STROBE) recommendations [15].

### 2.2. Participants

Singleton pregnancies between 24 + 0 and 36 + 6 weeks’ gestation were eligible. The preterm labor group comprised patients diagnosed clinically with preterm labor at presentation. The control group comprised asymptomatic pregnant women without a diagnosis of preterm labor at enrollment and was matched to cases by gestational age at ultrasound examination. Exclusion criteria included multiple gestation, major fetal structural or chromosomal anomalies, maternal systemic disease, and pregnancy complications expected to independently influence fetal hemodynamics or timing of delivery. All participants were followed until delivery. In the control group, 11/30 (36.7%) delivered at ≥37 + 0 weeks; we therefore performed a sensitivity analysis restricted to term deliveries (≥37 + 0 weeks) to assess the robustness of group differences.

Specifically, exclusion criteria included maternal systemic disease, chronic medication use, alcohol and smoking, and the development during follow-up of additional pregnancy complications such as preeclampsia or clinical chorioamnionitis, in accordance with the study protocol.

### 2.3. Definitions and Clinical Management

Preterm labor was defined according to contemporary clinical criteria, including regular uterine contractions with cervical change or progressive cervical dilatation, consistent with standard guidance [3]. Clinical management (including the use of tocolysis and antenatal corticosteroids) followed institutional protocols and was not dictated by study participation.

### 2.4. Ultrasound Examination and Measurements

All ultrasound examinations were performed using a Ultrasound examinations were performed using a Voluson E8 ultrasound system (GE Healthcare, Zipf, Upper Austria, Austria) equipped with the Voluson system software (GE Healthcare, Zipf, Upper Austria, Austria). Available at: https://www.gehealthcare.com/products/ultrasound/voluson. Accessed: 31 January 2026. Gestational age was determined by best obstetric estimate.

Transvaginal cervical length was measured with an empty bladder using standardized technique, recording the shortest of at least three measurements.

For fetal adrenal assessment, the fetal adrenal gland was identified adjacent to the fetal kidney in a transverse plane. Three orthogonal diameters (length, width, and depth) were measured. Fetal adrenal gland volume (AGV) was calculated using the ellipsoid formula (length × width × depth × 0.523) and expressed in cm^3^ [6]. Fetal zone depth was measured as the maximal width of the central fetal zone within the gland on the same standardized plane and expressed in millimeters [5,16,17]. Estimated fetal weight (EFW) at the index scan was obtained from the ultrasound report. Corrected adrenal gland volume (cAGV) was calculated as AGV/EFW and expressed in cm^3^/kg. A fetal zone–total gland depth ratio (FZD/AGD; d/D ratio) was calculated as fetal zone depth (FZD) divided by total adrenal gland depth (AGD).

For Doppler assessment, color Doppler was used to identify the middle adrenal artery supplying the fetal zone. Pulsed-wave Doppler was obtained with the smallest feasible sample volume and an insonation angle as close to 0° as possible (≤30°). At least three consecutive uniform waveforms were recorded, and pulsatility index (PI) was measured using automated tracing [13,18,19].

### 2.5. Outcomes

Primary outcomes were gestational age at delivery and neonatal intensive care unit admission. Secondary outcomes included birthweight and Apgar scores at 1 and 5 min.

### 2.6. Sample Size

An a priori sample size calculation was performed using G*Power 3.1 [20], informed by effect sizes reported in prior studies evaluating fetal adrenal markers in symptomatic preterm birth [7,11]. A minimum of 30 participants per group was targeted. In the study protocol, the a priori power analysis was performed in G*Power 3.1.9.7 (Düsseldorf, Germany) assuming a two-sample *t*-test (two-sided), α = 0.05, power (1 − β) = 0.95, and Cohen’s d = 1.71 (based on Ali et al. [11]), yielding a minimum of 10 participants per group (total N = 20). To ensure adequate power for prespecified subgroup analyses (e.g., antenatal corticosteroid exposure; late preterm vs. term delivery) and to account for potential non-normal distributions, we targeted 30 participants per group (total N = 60).

### 2.7. Statistical Analysis

Normality was assessed using the Shapiro–Wilk test. Continuous variables are presented as median (interquartile range) and categorical variables as n (%). Between-group comparisons used Mann–Whitney U tests for continuous variables and Fisher’s exact test for categorical variables. Odds ratios (OR) with 95% confidence intervals (CI) were calculated for NICU admission. Spearman correlation coefficients were used to evaluate associations between gestational age at delivery and ultrasound markers within the preterm labor group. A two-sided *p* value < 0.05 was considered statistically significant. As a sensitivity analysis, key comparisons were repeated after restricting the control group to term deliveries (≥37 + 0 weeks). In addition, cAGV (AGV/EFW) and the fetal zone–total gland depth ratio (FZD/AGD) were derived and analyzed using the same between-group comparisons and correlation approach.

### 2.8. Ethics

Ethics approval was obtained from the institutional ethics committee (Ankara Etlik City Hospital No. 1 Clinical Research Ethics Committee, Approval No: AEŞH-EK1-2023-476, Date: 16 August 2023). Written informed consent was obtained from all participants.

## 3. Results

A total of 60 participants were included (30 preterm labor, 30 controls). Gestational age at ultrasound was identical between groups (median 31 + 6 weeks in both), and maternal age did not differ significantly (Table 1). All participants had complete follow-up to delivery. Among controls, 11/30 (36.7%) delivered at ≥37 + 0 weeks.

Compared with controls, the preterm labor group had markedly shorter cervical length (median 12.5 vs. 33.5 mm, *p* < 0.001). Fetal adrenal gland volume and fetal zone depth were significantly higher in the preterm labor group (AGV 1.53 vs. 1.08 cm^3^; FZ 7.45 vs. 5.30 mm; both *p* < 0.001). Middle adrenal artery PI was lower in the preterm labor group (1.11 vs. 1.46, *p* < 0.001) (Table 1). In a sensitivity analysis restricted to term controls (≥37 + 0 weeks; n = 11), group differences in cervical length, AGV, fetal zone depth, and MAA-PI remained significant (all *p* < 0.001). EFW at the index scan was similar between groups (*p* = 0.900). Size-adjusted cAGV remained significantly higher in the preterm labor group (0.87 (0.76–1.06) vs. 0.59 (0.51–0.70), *p* < 0.001), and the fetal zone–total gland depth ratio (FZD/AGD) was also higher (0.328 (0.312–0.346) vs. 0.263 (0.241–0.278), *p* < 0.001).

Pregnancies in the preterm labor group delivered earlier (median 33 + 0 vs. 36 + 2 weeks, *p* < 0.001), had lower birthweight (median 1875 vs. 3188 g, *p* < 0.001), and lower Apgar scores (Table 1). NICU admission was more frequent in the preterm labor group (50.0% vs. 6.7%; OR 14.0, 95% CI 2.82–69.56; *p* < 0.001).

Within the preterm labor group, gestational age at delivery correlated positively with cervical length (ρ = 0.900, *p* < 0.001) and MAA-PI (ρ = 0.770, *p* < 0.001) and negatively with AGV (ρ = −0.770, *p* < 0.001) and fetal zone depth (ρ = −0.733, *p* < 0.001) (Table 2). Birthweight and Apgar scores were also positively correlated with gestational age at delivery. Using size-adjusted adrenal metrics, gestational age at delivery was strongly negatively correlated with cAGV (ρ = −0.953, *p* < 0.001) and with FZD/AGD (ρ = −0.733, *p* < 0.001).

## 4. Discussion

This study demonstrates a coherent adrenal signature in pregnancies presenting with preterm labor at the same gestational age as asymptomatic controls: larger fetal adrenal gland volume, wider fetal zone, and reduced middle adrenal artery pulsatility index. These differences were accompanied by earlier delivery and worse neonatal outcomes, including a markedly higher rate of NICU admission. Within the preterm labor group, adrenal enlargement and lower MAA-PI were strongly associated with earlier delivery, suggesting that both adrenal morphology and perfusion track with the intensity of the pathophysiologic process leading to preterm birth. Neonatal outcomes are primarily driven by gestational age at delivery; therefore, our findings should be interpreted as associations with earlier delivery rather than direct causal effects of adrenal markers on neonatal morbidity. A key contribution of our work is the combined assessment of adrenal morphology (AGV and fetal zone depth) and middle adrenal artery Doppler (MAA-PI) in a symptomatic triage setting.

Our findings are consistent with the concept proposed by Turan and colleagues that fetal adrenal gland changes reflect activation of the fetal hypothalamic–pituitary–adrenal axis and can precede spontaneous preterm birth. In symptomatic cohorts, fetal adrenal gland volume and fetal zone enlargement have shown good performance for identifying delivery within days [4,5]. Their methodological work comparing 2D and 3D volumetry supported feasibility and suggested that adrenal measures can be standardized sufficiently for clinical research [6]. In our study, AGV and fetal zone depth differed substantially between groups and correlated strongly with gestational age at delivery, supporting the biological plausibility of adrenal enlargement as a marker of imminent delivery.

Other studies have suggested that adrenal markers may approach the predictive value of cervical length for short-term outcomes. Lemos et al. reported that central fetal zone depth predicted delivery within 7 days with accuracy comparable to cervical length [7]. Costa Carvalho et al. showed that fetal adrenal artery Doppler indices predicted delivery within 7 days and that this prediction was similar to that obtained using cervical length [8]. While our study did not define a short-term prediction window, the strong associations of AGV, fetal zone depth, and MAA-PI with gestational age at delivery are consistent with the idea that adrenal morphology and perfusion change as birth approaches.

Multimodal approaches may be particularly relevant for clinical implementation. Agarwal et al. combined adrenal biometry with cervical elastography and reported improved prediction compared with single-modality assessment [9]. Santipap and Phupong combined 3D adrenal gland enlargement with placental alpha microglobulin-1 to improve sensitivity for delivery within 7 days in threatened preterm labor [10]. In addition, Ali et al. evaluated adrenal gland volume and blood flow in a pilot study and supported the feasibility of integrating perfusion assessment into prediction frameworks [11]. Taken together with our data, these studies suggest that combining cervical metrics with adrenal size and adrenal blood flow may help refine triage in symptomatic women.

A key contribution of our study is the inclusion of middle adrenal artery Doppler. The fetal adrenal gland receives zone-specific blood supply, with the middle adrenal artery primarily supplying the fetal zone, which is highly active in steroidogenesis. Reference-range work has clarified longitudinal patterns of inferior and middle adrenal artery Doppler indices and highlighted zonal differences, providing a foundation for standardized acquisition [13]. Recent feasibility work also demonstrated reliable visualization and velocity assessment of fetal adrenal arteries with modern ultrasound equipment [14]. In this context, our finding of lower MAA-PI in preterm labor and a strong positive association between MAA-PI and gestational age at delivery supports a physiologically coherent interpretation: reduced vascular resistance may reflect increased adrenal perfusion as part of the fetal stress response.

Recent studies have continued to explore adrenal-based markers using different imaging and study designs. A case–control study using volumetric adrenal measurements reported significant differences between preterm labor and controls and suggested predictive value for preterm delivery [15]. An MRI-based pilot study in fetuses delivering before 32 weeks highlighted the complexity of adrenal size metrics and suggested that ratios (such as adrenal-to-body volume) may provide complementary information [16]. These observations emphasize that adrenal metrics may depend on measurement technique and fetal size, and they support further methodological work to identify the most reproducible and clinically informative adrenal parameters.

This study has limitations. It was conducted at a single center with a modest sample size. Controls were defined as asymptomatic at enrollment rather than by term delivery, which may reduce contrast compared with a strictly term control group. We performed post hoc fetal size adjustment by retrieving EFW from the ultrasound report and calculating corrected adrenal gland volume (cAGV) and the fetal zone–total gland depth ratio (FZD/AGD); however, abdominal circumference was not consistently available and EFW was retrieved retrospectively. Inter- and intraobserver reproducibility were not assessed. Finally, the analysis was primarily univariable and did not evaluate multivariable models or predefined time-to-delivery thresholds. These limitations should be addressed in larger prospective cohorts that focus on clinically relevant prediction horizons, standardized acquisition, and evaluation of incremental value beyond cervical length and biochemical tests. Notably, because a substantial proportion of controls delivered <37 weeks, we performed a sensitivity analysis restricted to term controls (≥37 + 0 weeks), which yielded consistent findings.

Future directions: Larger multicenter prospective studies should (i) evaluate adrenal markers earlier in gestation and as longitudinal trajectories, (ii) incorporate standardized intra- and interobserver reproducibility assessment, (iii) report fetal size-adjusted adrenal metrics, and (iv) test whether integrating adrenal morphology and Doppler indices improves short-term prediction horizons and clinical decision-making beyond cervical length and biochemical markers.

In conclusion, pregnancies presenting with preterm labor showed enlarged fetal adrenal gland volume and fetal zone depth and reduced middle adrenal artery PI compared with asymptomatic gestational age-matched controls. These markers were strongly associated with gestational age at delivery and adverse neonatal outcomes, supporting their potential role as adjunct sonographic markers in the assessment of symptomatic preterm labor.

## 5. Conclusions

Fetal adrenal gland volume, fetal zone depth, and middle adrenal artery pulsatility index differed significantly between pregnancies with preterm labor and asymptomatic controls at the same gestational age. Larger adrenal measures and lower MAA-PI were associated with earlier delivery and higher NICU admission. These findings support further prospective evaluation of combined adrenal morphology and Doppler assessment for short-term risk stratification in symptomatic preterm labor.

## Figures and Tables

**Table 1:** Participant characteristics and ultrasound markers.

Variable	Preterm Labor (n = 30)	Controls (n = 30)	*p* Value
Maternal age (years)	28 (26–32)	33 (27–36)	0.063
Gestational age at ultrasound (weeks)	31 + 6 (30 + 5–33 + 0)	31 + 6 (30 + 5–33 + 0)	1.000
Estimated fetal weight, EFW (g)	1740 (1550–2000)	1775 (1550–2022)	0.900
Cervical length (mm)	12.5 (10.0–15.0)	33.5 (31.2–36.0)	<0.001
Fetal adrenal gland volume, AGV (cm^3^)	1.53 (1.46–1.67)	1.08 (1.01–1.12)	<0.001
Corrected adrenal gland volume, cAGV (cm^3^/kg)	0.87 (0.76–1.06)	0.59 (0.51–0.70)	<0.001
Fetal zone depth (mm)	7.45 (7.00–8.00)	5.30 (4.90–5.57)	<0.001
Fetal zone–total gland depth ratio (FZD/AGD)	0.328 (0.312–0.346)	0.263 (0.241–0.278)	<0.001
Middle adrenal artery PI (MAA-PI)	1.11 (1.02–1.20)	1.46 (1.38–1.51)	<0.001
Gestational age at delivery (weeks)	33 + 0 (31 + 1–34 + 0)	36 + 2 (35 + 4–37 + 5)	<0.001
Birthweight (g)	1875 (1600–2050)	3188 (2986–3426)	<0.001
Apgar score at 1 min	6 (4–7)	8 (8–8)	<0.001
Apgar score at 5 min	8 (7–9)	9 (8–10)	0.001
NICU admission, n/N (%)	15/30 (50.0%)	2/30 (6.7%)	<0.001

Values are median (interquartile range) unless otherwise specified. Gestational age is shown as weeks + days. *p* values are from Mann–Whitney U tests or Fisher’s exact test.

**Table 2:** Correlations with gestational age at delivery in the preterm labor group (n = 30).

Variable	Spearman Rho	*p* Value
Cervical length (mm)	0.900	<0.001
AGV (cm^3^)	−0.770	<0.001
cAGV (cm^3^/kg)	−0.953	<0.001
Fetal zone depth (mm)	−0.733	<0.001
FZD/AGD ratio	−0.733	<0.001
MAA-PI	0.770	<0.001
Birthweight (g)	0.976	<0.001
Apgar 1 min	0.859	<0.001
Apgar 5 min	0.838	<0.001

Spearman correlation coefficients (ρ).

## Data Availability

De-identified data may be made available by the corresponding author upon reasonable request, subject to institutional approvals.

## Abbreviations

AGD, adrenal gland depth; AGV, fetal adrenal gland volume; cAGV, corrected adrenal gland volume; CI, confidence interval; EFW, estimated fetal weight; FZ, fetal zone; FZD, fetal zone depth; GA, gestational age; MAA, middle adrenal artery; NICU, neonatal intensive care unit; OR, odds ratio; PI, pulsatility index.
